# Supplementary material for: The Reconstitution Dynamics of Cultivated Hematopoietic Stem Cells and Progenitors Is Independent of Age
Source: Int J Mol Sci. 2022 Mar 15;23(6):3160. doi: 10.3390/ijms23063160 (PMC8948791; doi:10.3390/ijms23063160)
Supplement: Supplementary file 1 [file ijms-23-03160-s001.zip › ijms-1620981-supplementary.pdf]

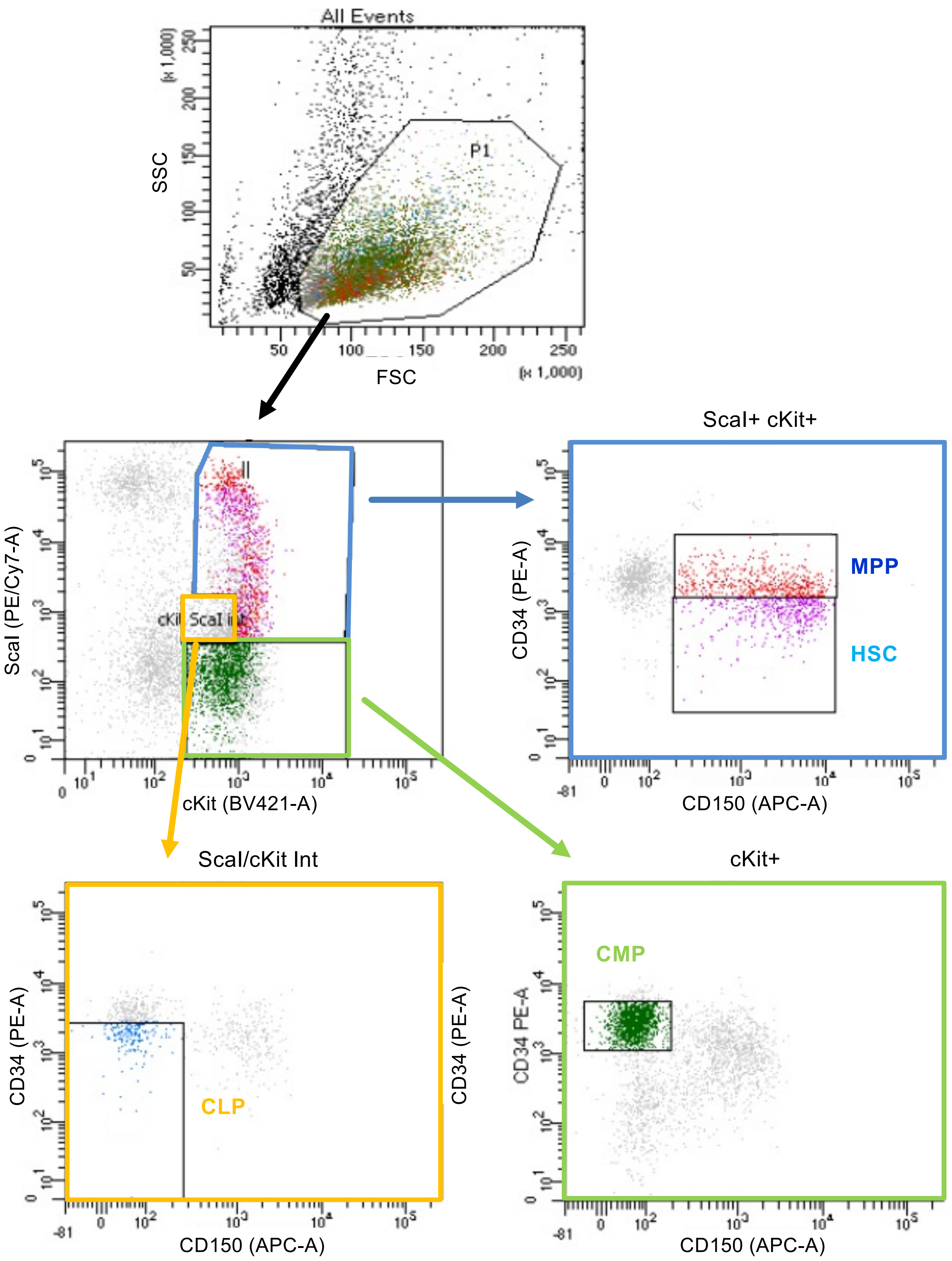

Supplementary Figure S1: Sorting scheme of HSPC subpopulations based on their surface marker profile. Bone marrow cells were harvested from male donor animals and after MACS-based lineage-depletion stained with the respective antibodies (Supplementary Table 1) against the markers ScaI, cKit, CD34 and CD150. HSCs were defined as ScaI+ cKit+ CD150+ CD34-,
MPPs as ScaI+ cKit+ CD150+ CD34+,
CMPs as ScaI- cKit+ CD150- CD34+ and
CLPs as ScaI/ cKit Int CD150- CD34-.

Colors of the plots show parental gate in the former plot and resemble the color scheme of HSPC subpopulation used for visualization of the stained population in the subsequent plots.

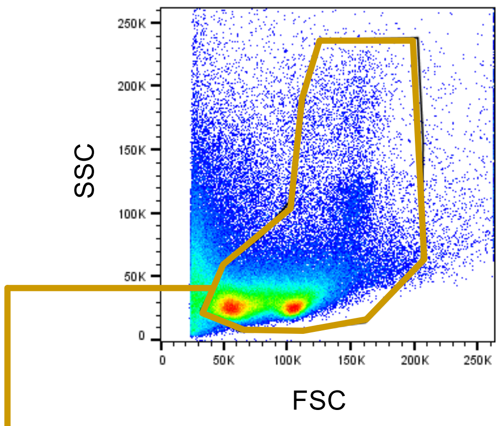

Supplementary Figure S2: Gating strategy to determine the frequency of initially marked HSPC in mature blood cell populations.
Single suspensions from spleen were first stained with antibodies against CD3e (for T cells), against B220 (for B cells) from the lymphoid compartment and against CD11b (for monocytes/macrophages and against CD11b and Ly6G (for granulocytes). After determination of the morphological cell population (P1), subsequent gating on the respective stained mature cell populations showed the frequency of the marked cells. Shown is one representative animal from the O-O group.

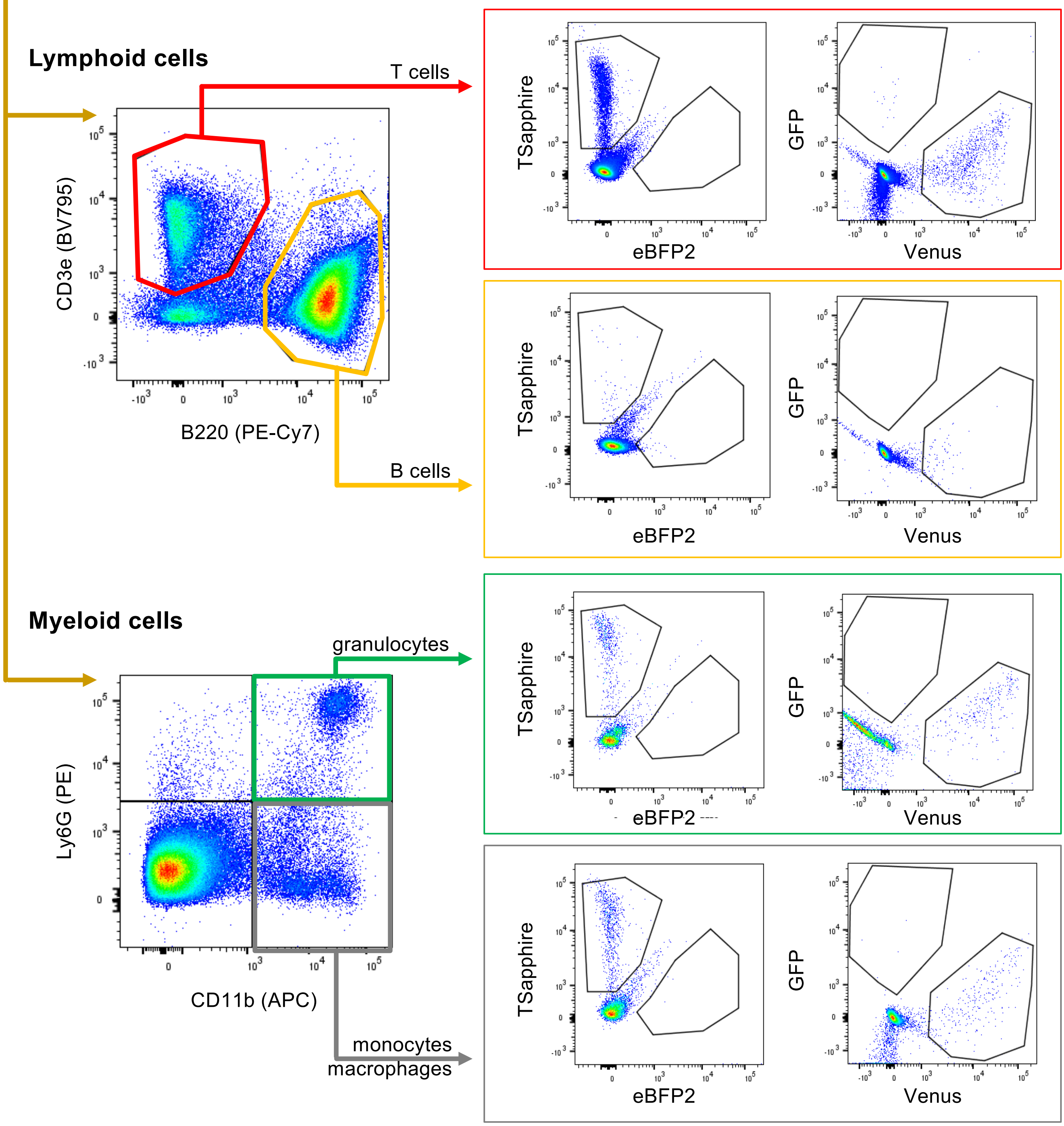

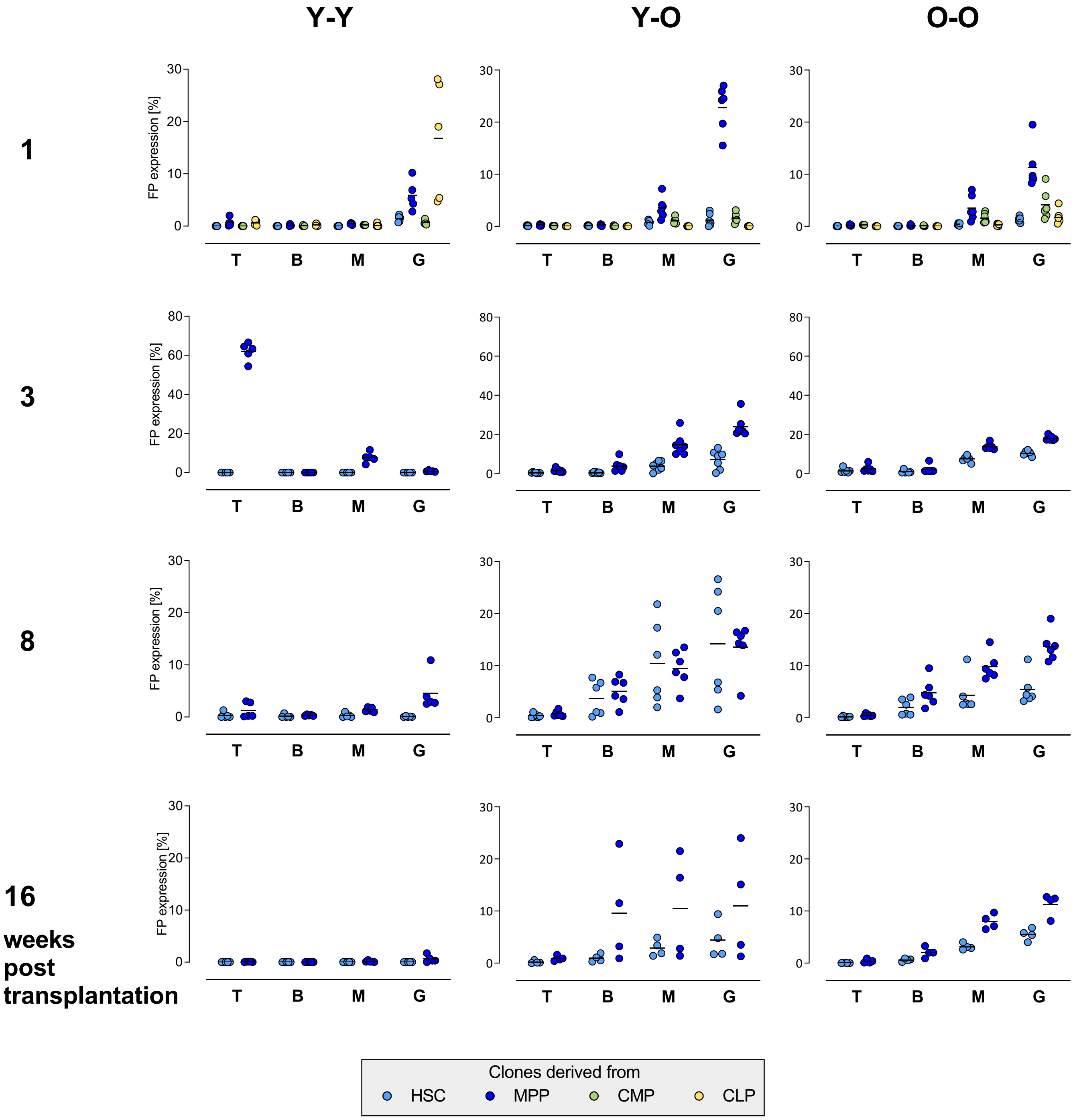

Supplementary Figure S3: Contribution of marked cell populations in mature blood cell compartments
FP expression was measured in the T cell (T; CD3), B cell (B; B220), monocyte/macrophage (M; CD11b) and the Granulocyte (G; CD11b/Ly6G) compartment by FACS analysis. The spleen was mostly comprised of HSC- and MPP-derived cells. CMP-derived cells were solely detectable 1 week after transplantation. CLP-derived cells were barely detectable. No data available for CMP- and CLP-derived cells 3, 8 or 16 weeks post transplantation.
Light-blue dots: HSC-derived clones, dark-blue dots: MPP-derived clones, green dots: CMP-derived clones and yellow dots: CLP-derived clones.

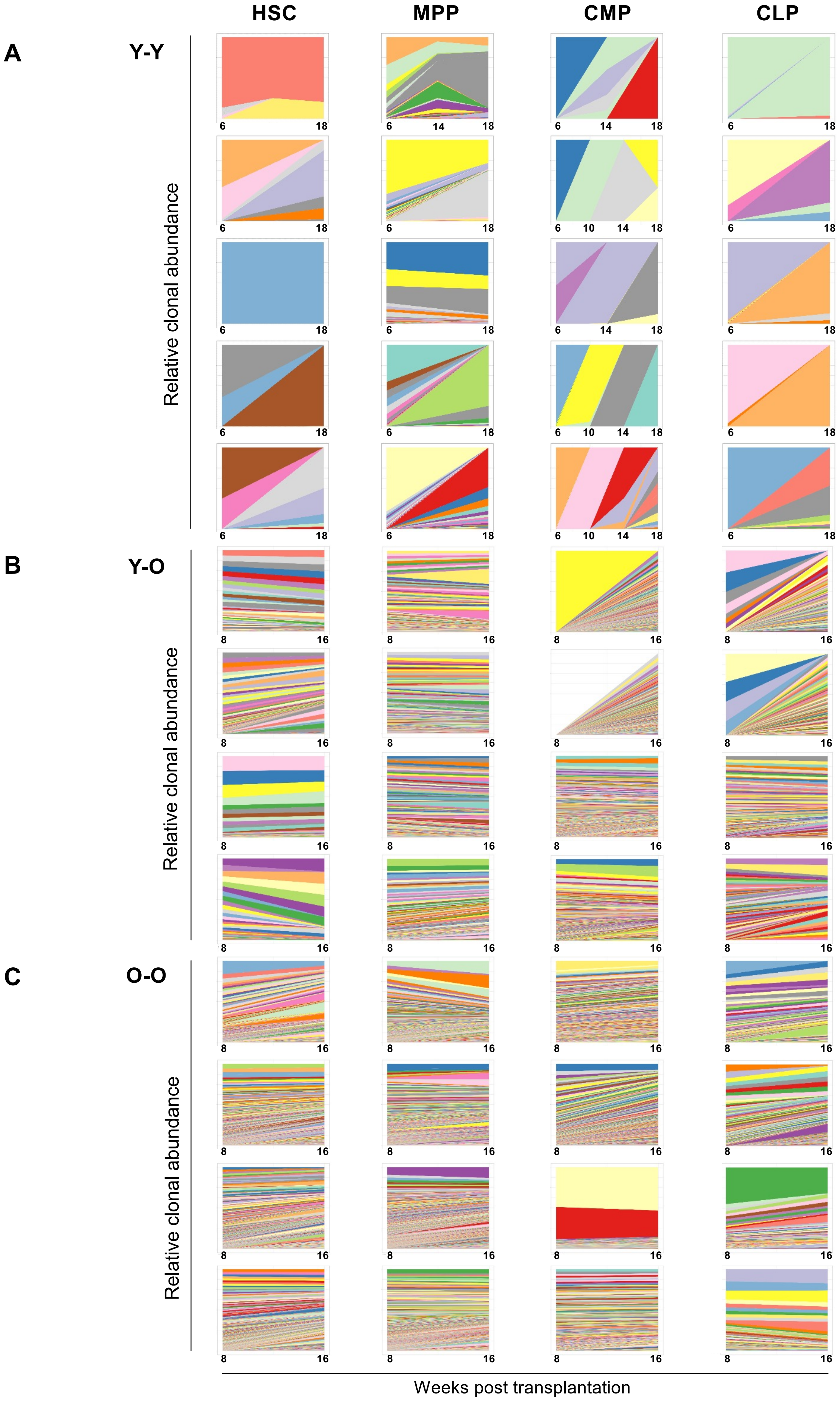
HSC
MPP
CMP
CLP
A
Y-Y
B
Y-O
C
O-O
Relative clonal abundance
Weeks post transplantation

Supplementary Figure S4: Contribution of unique clones in peripheral blood.

Consecutive peripheral blood samples were harvested from animals of the Y-Y, Y-O and O-O experimental groups throughout the experimental course. For the Y-Y group, up to four samples were taken on week 6, 10, 14 and 18 post transplantation. For the Y-O and O-O group, two samples at 8 or 16 weeks post transplantation were taken. Depending on the amount of obtained blood, the concentration of the extracted DNA was too low to perform PCR based barcode-retrieval. Therefore, we omitted those data points from the plots.

Relative clonal abundancies are depicted as a function of the measurement time points. Each clone is shown with one individual color. An overall lower abundancy of clones was observed in the Y-Y experimental part, while Y-O and O-O experimental parts were more heterogeneous. Each row of plots represents one representative animal from each group.

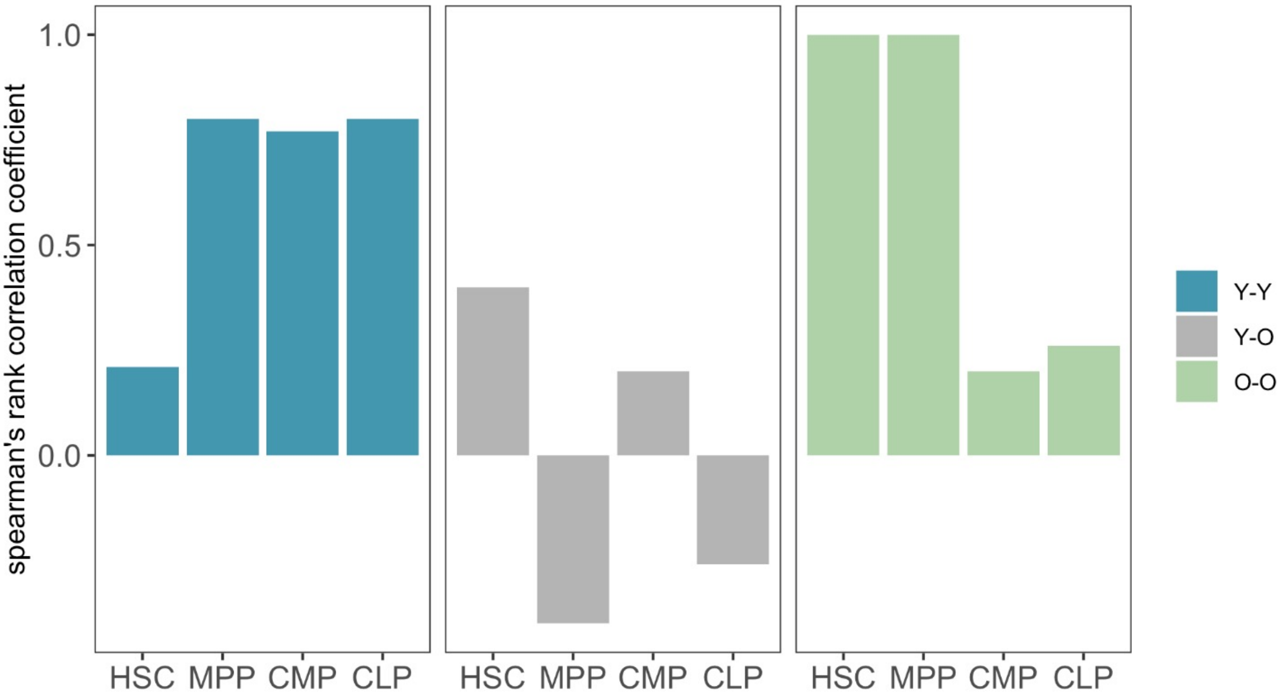

Supplementary Figure S5: Spearman's correlation coefficients to quantify the correlation between flow cytometry and barcode data
The mean values of the percentage of fluorescent positive cells taken from spleen (Fig. 4) at each time point and for each experimental setting and the respective mean barcode counts (Fig. 5) were used to calculate the Spearman's rank correlation coefficients to quantify the interrelation of the two measurement methods.

Supplementary Table S1: Antibodies used for fluorescence-activated cell sorting of HSPC populations and final analyses.

Name	Clone	Company	Application
anti CD16/32	93	Biolegend	Sort of HSPC subpopulations/ Final Analyses
PE-Cy7 anti ScaI	E13-161.7	Biolegend	Sort of HSPC subpopulations/ Final Analyses of BM populations
BV421 anti cKit	2B8	BD Biosciences	Sort of HSPC subpopulations
Pacific Blue anti cKit	2B8	Biolegend	Sort of HSPC subpopulations
APC anti CD150	TC15-12F12.2	Biolegend	Sort of HSPC subpopulations
PE anti CD34	RAM34	BD	Sort of HSPC subpopulations
APC-Cy7 anti cKit	2B8	Biolegend	Final Analyses of BM populations
PE anti CD150	TC15-12F12.2	Biolegend	Final Analyses of BM populations
APC anti lineage	diverse	BD (558074)	Final Analyses of BM populations
APC anti lineage	diverse	R&D Systems (FLC001A)	Final Analyses of BM populations
APC anti CD48	HM48-1	Biolegend	Final Analyses of BM populations
PE-Cy7 anti B220	RA3-6B2	Biolegend	Final analyses of mature blood cells in spleen
BV795 anti CD3e	145-2C11	BD	Final analyses of mature blood cells in spleen
PE anti Ly.6G	1A8	Biolegend	Final analyses of mature blood cells in spleen
APC anti CD11b	M1/70	Biolegend	Final analyses of mature blood cells in spleen

Supplementary Table S2: Oligonucleotides used for Barcode library construction and analysis.
N positions indicate sites for random insertion of one of the four nucleotides during production, x indicates phosphothioate bond and BHQ1 stands for Black Hole Quencher 1.

Internal name	Sequence (5‘-3‘)	Application
Poly-GFP-BC-fw	GGTGCATCTAGAACACTCTTTCCCTACACGACGCTCTTCCGATCTNNNACTNNCGANNCTTNNCGANNCTTNNGGANNCTANNACTNNCGANNCTTNNCGANNCTTNNGGANNCTANNACTNNCGANNCTCGAGGTGCACTATG	Oligo for GFP-BC library construction
Poly-Venus-BC-Fw	GGTGCATCTAGAACACTCTTTCCCTACACGACGCTCTTCCGATCTNNNCGANNAGANNCTTNNCGANNCTANNGGANNCTTNNCGANNAGANNCTTNNCGANNCTANNGGANNCTTNNCGANNAGANNCTCGAGGTGCACTATG	Oligo for Venus-BC library construction
Poly-Cerulean-BC-Fw	GGTGCATCTAGAACACTCTTTCCCTACACGACGCTCTTCCGATCTNNNCAGNNATCNNCTTNNCGANNGGANNCTANNCTTNNCAGNNATCNNCTTNNCGANNGGANNCTANNCTTNNCAGNNATCNNCTCGAGGTGCACTATG	Oligo for T-Sapphire-BC library construction
Poly-Cherry-BC-Fw	GGTGCATCTAGAACACTCTTTCCCTACACGACGCTCTTCCGATCTNNNCTANNCAGNNCTTNNCGANNCTANNCTTNNGGANNCTANNCAGNNCTTNNCGANNCTANNCTTNNGGANNCTANNCAGNNCTCGAGGTGCACTATG	Oligo for BFP-BC library construction
32BC-Poly-fw	GGTGCATCTAGAACACTC	Generation of ds BC oligo
32BC-Poly-rev	CATAGTGCACCTCGAG	Generation of ds BC oligo
Illu_P2 (43)	GTGACTGGAGTTCAGACGTGTGCTCTTCCGATCT	Bridging oligo for sequencing
Illu_MPLX35 (164)	CAAGCAGAAGACGGCATACGAGATTCTGAGGTGACTGGAGTTC	Index35
Illu_MPLX36 (165)	CAAGCAGAAGACGGCATACGAGATCCTTGCGTGACTGGAGTTC	Index36
Illu_MPLX37 (166)	CAAGCAGAAGACGGCATACGAGATTGGAGCGTGACTGGAGTTC	Index37
Illu_MPLX38 (167)	CAAGCAGAAGACGGCATACGAGATTCGGGAGTGACTGGAGTTC	Index38
Illu_MPLX39 (168)	CAAGCAGAAGACGGCATACGAGATAAACCT GTGACTGGAGTTC	Index39
Illu_MPLX40 (169)	CAAGCAGAAGACGGCATACGAGAT CTCTAC GTGACTGGAGTTC	Index40
Illu_MPLX41 (170)	CAAGCAGAAGACGGCATACGAGAT CGGCCT GTGACTGGAGTTC	Index41
Illu_MPLX42 (171)	CAAGCAGAAGACGGCATACGAGATCCGGTGGTGACTGGAGTTC	Index42
Illu_MPLX43 (172)	CAAGCAGAAGACGGCATACGAGATCAGCAGGTGACTGGAGTTC	Index43
Illu_MPLX44 (173)	CAAGCAGAAGACGGCATACGAGATAAGTGCGTGACTGGAGTTC	Index44
Illu_MPLX45 (174)	CAAGCAGAAGACGGCATACGAGATCAGGCCGTGACTGGAGTTC	Index45
Illu_MPLX46 (175)	CAAGCAGAAGACGGCATACGAGATGGTAGAGTGACTGGAGTTC	Index46
Illu_MPLX47 (176)	CAAGCAGAAGACGGCATACGAGATCCAGCAGTGACTGGAGTTC	Index47
Illu_MPLX48 (177)	CAAGCAGAAGACGGCATACGAGATGCGCCAGTGACTGGAGTTC	Index48
Illu_MPLX49 (178)	CAAGCAGAAGACGGCATACGAGATGGAACTGTGACTGGAGTTC	Index49
Illu_MPLX50 (179)	CAAGCAGAAGACGGCATACGAGATGCGGACGTGACTGGAGTTC	Index50
Illu_MPLX51 (180)	CAAGCAGAAGACGGCATACGAGATCGAAACGTGACTGGAGTTC	Index51
Illu_MPLX52 (181)	CAAGCAGAAGACGGCATACGAGATCCACTCGTGACTGGAGTTC	Index52
ILL_Dual_P5-01	AATGATACGGCGACCACCGAGATCTACACAGCTTAGTACACTCTTTCCCTACACGACGCTCTTCCGATCxT	Dual Index01
ILL_Dual_P5-02	AATGATACGGCGACCACCGAGATCTACACGCTACTTGACACTCTTTCCCTACACGACGCTCTTCCGATCxT	Dual Index02
ILL_Dual_P5-03	AATGATACGGCGACCACCGAGATCTACACCTAGGCACACACTCTTTCCCTACACGACGCTCTTCCGATCxT	Dual Index03
ILL_Dual_P5-04	AATGATACGGCGACCACCGAGATCTACACTAGCAGCAACACTCTTTCCCTACACGACGCTCTTCCGATCxT	Dual Index04
Y Chromosome Fw	ACAAGTTTTGGGACTGGTGACAA	ddPCR Y chromosome
Y Chromosome Rv	ACCACGGGACCACACCATAA	ddPCR Y chromosome
Y Chromosome-p	TGTCAAGCGCCCCATGAATGCA-BHQ1	ddPCR Y chromosome
LeGOddBCi Fw	CTAGAAAAACATGGAGCAATCACAA	ddPCR illumina sequence
ddBCillu Rv	AGCGTCGTGTAGGGAAAGAGTG	ddPCR illumina sequence
ddBCIllu-p	CTGAATGATACGGCGACCACCGTCTAG	ddPCR illumina sequence
mEpo Chromosome Fw	GCAGGCGGGGTCGCTACTC	ddPCR mEpo
mEpo Chromosome Rv	CGCCTGTGCAGATCCGATAA	ddPCR mEpo
mEpo Chromosome-p	TTCTGAGGCGCCACTTTTGCAAGACC	ddPCR mEpo

Supplementary Table S3: Indirect estimation of the average vector copy number

Single cells from spleen of transplanted animals (randomly chosen from the respective experimental group) were sorted based on their fluorescent protein expression. Cells were directly used for PCR to amplify the barcode sequence(s) using wpre-fw (5'-GAGGAGTTGTGGCCCGTTGT) and TA98 (5'-GGCTGTCAAACCTCCACTCT) for the first PCR and TA97 (5'-CATCGATACCGTCGACCTC) and TA99 (5'-TCGTTGGGAGTGAATTAGCC) for the second PCR. PCR-products were gelpurified and Sanger sequenced using TA99. Individual vector copy numbers (VCN) were determined as described in Bystrykh et al., 2016. Assuming that the viral integrations per cell are independent events, the total number of integrations per cell (individual vector copy number, VCN) follows a Poisson distribution. Based on the frequency of individual VCN observed among the single cells, we fit such a Poisson distribution to the empirical data for which the parameter lambda corresponds to the mean number of integrations (examples below the table for the HSC derived samples). Based on these fits we can also estimate the fraction of "untransduced cells" which is given as exp(-lambda). Conversely, the fraction of marked cells (i.e. the transduction efficiency) can be calculated as (1 - exp(-lambda)).

Y-O				O-O			
HSC-derived		MPP-derived		HSC-derived		MPP-derived	
Sample	VCN	Sample	VCN	Sample	VCN	Sample	VCN
1	3.0	1	3.0	1	2.0	1	2.0
2	3.5	2	3.0	2	2.0	2	2.0
3	5.0	3	5.5	3	2.0	3	4.0
4	2.0	4	3.5	4	1.0	4	3.0
5	2.0	5	4.0	5	1.0	5	5.5
6	3.0	6	4.0	6	1.0	6	2.0
7	2.0	7	2.0	7	4.5	7	3.0
8	3.0	8	2.0	8	1.0	8	1.0
9	1.0	9	2.5	9	1.0	9	2.0
10	3.0	10	2.5	10	1.0	10	2.0
11	2.0	11	3.0	11	2.0	11	5.0
12	4.0	12	3.5	12	2.0	12	2.0
13	4.0	13	3.0	13	2.0	13	1.0
14	4.0	14	5.5	14	2.0	14	1.0
15	1.0	15	1.0	15	3.0	15	1.0
16	6.0	16	5.0	16	2.0	16	1.0
17	1.0	17	4.0	17	4.0	17	2.0
18	4.0	18	6.0			18	2.0
19		19	1.0			19	3.0
20		20	1.0			20	1.0
21		21	3.0				
mean	2.8	mean	3.2	mean	1.6	mean	2.4

Y–O, HSC, mean of poisson = 2.821 , fraction labeled = 0.94

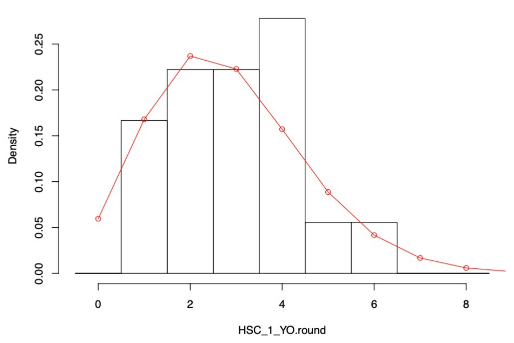

O–O, HSC, mean of poisson = 1.594 , fraction labeled = 0.797

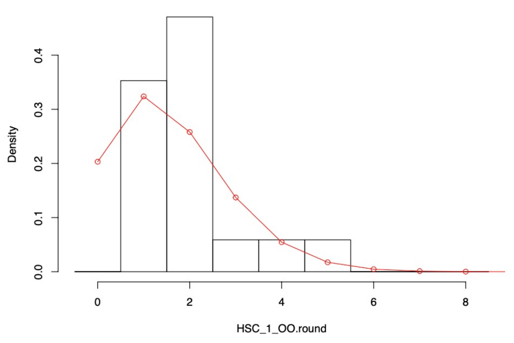

Supplementary Table S4: Fluorescent protein expressing cells in the mature blood cells
Flow cytometry data from spleen of the animals (n= 5-7) per group taken after 1,3, 8 or 16 weeks post transplantation. The gating strategy is shown in Supplementary Figure 2. Listed are the mean values and the respective range (min-max).

			T cells		B cells		Mono / Macro		Granulocytes	
	week	ini. subp.	%	min-max	%	min-max	%	min-max	%	min-max
Y-Y	1	HSC	0,0	0,0-0,0	0,0	0,0-0,0	0,0	0,0-0,0	1,4	0,7-2,2
		MPP	0,1	0,0-0,4	0,7	0,1-2,0	0,4	0,2-0,6	5,9	2,8-10,2
		CMP	0,1	0,0-0,2	0,0	0,0-0,0	0,2	0,2-0,3	0,7	0,3-1,4
		CLP	0,2	0,0-0,5	0,7	0,1-1,2	0,1	0,0-0,7	16,9	4,7-28,1
	3	HSC	0,0	0,0-0,0	0,0	0,0-0,0	0,0	0,0-0,0	0,0	0,0-0,0
		MPP	0,0	0,0-0,0	62,0	54,4-66,6	7,7	4,2-11,6	0,7	0,4-1,3
	8	HSC	0,2	0,0-0,7	0,3	0,0-1,3	0,3	0,1-1,0	0,1	00-0,2
		MPP	0,3	0,2-0,5	1,3	0,1-3,0	1,4	0,9-1,8	4,6	2,5-10,9
	16	HSC	0,0	0,0-0,0	0,0	0,0-0,0	0,0	0,0-0,0	0,0	0,0-0,0
		MPP	0,0	0,0-0,0	0,1	0,0-0,1	0,1	0,0-0,4	0,6	0,0-1,7
Y-O	1	HSC	0,1	0,0-0,2	0,1	0,0-0,2	0,7	0,1-1,3	1,2	0,0-3,0
		MPP	0,2	0,1-0,4	0,2	0,0-0,4	3,6	1,2-7,2	22,8	15,5-27,0
		CMP	0,1	0,0-0,1	0,1	0,0-0,2	1,0	0,5-2,1	8,2	0,4-3,3
		CLP	0,0	0,0-0,0	0,0	0,0-0,0	0,0	0,0-0,0	0,0	0,0-0,0
	3	HSC	0,4	0,0-0,8	0,3	0,0-0,7	3,7	0,0-6,3	7,1	0,2-13,1
		MPP	1,5	0,7-3,3	3,7	1,3-9,8	14,5	9,9-25,8	23,9	20,4-35,6
	8	HSC	0,4	0,1-1,1	3,7	0,2-7,7	10,4	2,0-21,8	14,2	1,6-26,6
		MPP	0,8	0,3-1,7	5,1	1,1-8,3	9,5	3,7-10,8	13,5	4,2-16,7
	16	HSC	0,2	0,0-0,6	1,0	0,3-1,9	2,9	1,4-4,9	4,4	1,7-9,4
		MPP	0,9	0,4-1,6	9,6	0,9-22,9	10,5	1,4-21,5	11,0	1,3-24,0
O-O	1	HSC	0,0	0,0-0,1	0,0	0,0-0,1	0,4	0,1-0,6	1,3	0,6-2,1
		MPP	0,2	0,1-0,4	0,2	0,1-0,4	3,5	0,9-7,0	11,3	9,1-19,5
		CMP	0,3	0,2-0,3	0,1	0,0-0,2	1,6	0,7-2,9	4,1	1,4-9,1
		CLP	0,0	0,0-0,0	0,0	0,0-0,0	0,3	0,0-0,5	1,8	0,5-4,4
	3	HSC	1,4	0,4-3,7	0,8	0,4-2,3	7,4	4,9-9,5	10,4	8,3-12,0
		MPP	2,3	1,1-5,9	2,2	1,2-6,5	13,7	12,3-16,8	18,0	17,0-20,2
	8	HSC	0,2	0,1-0,4	2,0	0,6-3,5	4,3	2,5-11,2	5,4	3,2-11,2
		MPP	0,5	0,3-0,9	4,8	1,8-9,5	9,8	7,5-14,5	13,7	10,8-19,0
	16	HSC	0,0	0,0-0,1	0,6	0,2-0,9	3,2	2,6-4,0	5,5	4,0-6,8
		MPP	0,4	0,1-0,9	2,1	0,9-3,3	8,0	6,5-9,7	11,3	8,1-12,7
